# Preventive Analgesia, Hemodynamic Stability, and Pain in Vitreoretinal Surgery

**DOI:** 10.3390/medicina57030262

**Published:** 2021-03-12

**Authors:** Michał Jan Stasiowski, Aleksandra Pluta, Anita Lyssek-Boroń, Magdalena Kawka, Lech Krawczyk, Ewa Niewiadomska, Dariusz Dobrowolski, Robert Rejdak, Seweryn Król, Jakub Żak, Izabela Szumera, Anna Missir, Przemysław Jałowiecki, Beniamin Oskar Grabarek

**Affiliations:** 1 Department of Emergency Medicine, Faculty of Medical Sciences, Medical University of Silesia, 41-200 Sosnowiec, Poland; apluta@autograf.pl (A.P.); lech.kraw@gmail.com (L.K.); sernik7@gmail.com (J.Ż.); iza_sz@vp.pl (I.S.); aniami521@interia.pl (A.M.); olaf@pro.onet.pl (P.J.); 2 Department of Anaesthesiology and Intensive Therapy, 5th Regional Hospital, Medykow Square 1, 41-200 Sosnowiec, Poland; seweryn.krol@gmail.com; 3 Department of Ophthalmology with Paediatric Unit, 5th Regional Hospital, Medykow Square 1, 41-200 Sosnowiec, Poland; anitaboron3@gmail.com (A.L.-B.); mkawka.buszman@gmail.com (M.K.); 4 Department of Ophthalmology, Faculty of Medicine in Zabrze, University of Technology, 41-800 Zabrze, Poland; 5 Department of Epidemiology and Biostatistics, Faculty of Health Sciences, Medical University of Silesia, 41-902 Bytom, Poland; e.j.niewiadomska@gmail.com; 6 Chair and Clinical Department of Ophthalmology, Faculty of Medical Sciences, Medical University of Silesia, 41-200 Zabrze, Poland; dardobmd@wp.pl; 7 Department of General Ophthalmology, Medical University of Lublin, 20-059 Lublin, Poland; robert.rejdak@umlub.pl; 8 Department of General, Colorectal and Polytrauma Surgery, Faculty of Health Sciences, Medical University of Silesia, 40-055 Katowice, Poland; 9 Department of Histology, Cytophysiology and Embryology, Faculty of Medicine, University of Technology in Katowice, 41-800 Zabrze, Poland; bgrabarek7@gmail.com; 10 Department of Nursing and Maternity, High School of Strategic Planning, 41-300 Dąbrowa Górnicza, Poland

**Keywords:** vitreoretinal surgery, general anaesthesia, peribulbar block, topical anaesthesia, surgical pleth index

## Abstract

*Background and Objectives:* Although vitreoretinal surgery (VRS) is most commonly performed under regional anaesthesia (RA), in patients who might be unable to cooperate during prolonged procedures, general anaesthesia (GA) with intraprocedural use of opioid analgesics (OA) might be worth considering. It seems that the surgical pleth index (SPI) can be used to optimise the intraprocedural titration of OA, which improves haemodynamic stability. Preventive analgesia (PA) is combined with GA to minimise intraprocedural OA administration. *Materials and Methods:* We evaluated the benefit of PA combined with GA using SPI-guided fentanyl (FNT) administration on the incidences of PIPP (postprocedural intolerable pain perception) and haemodynamic instability in patients undergoing VRS (*p* < 0.05). We randomly assigned 176 patients undergoing VRS to receive GA with SPI-guided FNT administration alone (GA group) or with preventive topical 2% proparacaine (topical anaesthesia (TA) group), a preprocedural peribulbar block (PBB) using 0.5% bupivacaine with 2% lidocaine (PBB group), or a preprocedural intravenous infusion of 1.0 g of metamizole (M group) or 1.0 g of paracetamol (P group). *Results:* Preventive PBB reduced the intraprocedural FNT requirement without influencing periprocedural outcomes (*p* < 0.05). Intraprocedural SPI-guided FNT administration during GA resulted in PIPP in 13.5% of patients undergoing VRS and blunted the periprocedural effects of preventive intravenous and regional analgesia with respect to PIPP and haemodynamic instability. *Conclusions:* SPI-guided FNT administration during GA eliminated the benefits of preventive analgesia in the PBB, TA, M, and P groups following VRS.

## 1. Introduction

Vitreoretinal procedures (vitreoretinal surgery (VRS) or pars plana vitrectomy (PPV)) are most commonly performed under regional anaesthesia (RA) [1,2,3]. General anaesthesia (GA) is resorted to in those patients who may be unable to cooperate during prolonged procedures [4,5,6,7]. GA provides excellent intraprocedural immobilisation during VRS; however, the intraprocedural use of opioid analgesics (OA) during GA has been identified. It should be considered as an independent risk factor for the occurrence of postoperative nausea and vomiting (PONV) [8]. Therefore, these agents should be best avoided if possible [8]. It is indicated that they are necessary if there are signs of insufficient intraprocedural analgesia, such as hypertension or tachycardia, which may lead to cardiac and cerebrovascular events [9]. However, on the other hand, research by Loring et al. confirmed that low-dose intraoperative opioids reduce postprocedural pain without side-effects [10]. 

OA dosing during GA is currently defined by the observation of haemodynamic parameters linked with the clinical judgement of the anaesthesiologist [11]. However, insufficient intraprocedural analgesia might not necessarily lead to both tachycardia and hypertension as volatile anaesthetic agents tend to blunt the haemodynamic response to nociceptive stimulation [11]. The absence of a haemodynamic response might be more common either in diabetic patients or in older patients with sick sinus syndrome and may lead to intolerable pain perception after surgery [12].

For this reason, in the administration of OA, intraprocedural monitoring of analgesia using the analgesia nociception index, the surgical pleth index (SPI), and pupillometry is rising in popularity [13]. SPI has recently been introduced to titrate OA dosing during GA [13,14]. The SPI reflects a nociception–anti-nociception balance. It is believed that that SPI-guided analgesia provides better titration of the OA dose compared to observation of the haemodynamic response to intraprocedural painful stimuli [15]. The SPI has been indicated to be a better measure of the nociception–anti-nociception balance in comparison with either heart rate (HR) or blood pressure variations [16,17]. Moreover, differences in the SPI value after a bolus administration of fentanyl (FNT) allow for the monitoring and titration of intraprocedural dosing [18]. Rational titration of rescue doses of OA using SPI guidance can reduce the cumulative dose of narcotic analgesics during GA [19]. Changes in the SPI value in response to painful stimuli have been indicated to depend on the concentration of opioids in patients’ serum [20]. The utility of intraprocedural FNT titration guided via monitoring of analgesia has also been demonstrated to decrease postprocedural intolerable pain perception (PIPP) in comparison with standard practice [21]. 

Attempts have been made to reduce the dose of intraprocedural OA via different techniques of preventive analgesia for VRS, among which the most popular are regional techniques, including the preprocedural peribulbar block (PBB) [22,23,24,25,26], retrobulbar block (RBB) [27,28], subtendon block [29,30], and topical anaesthesia (TA) [31], as well as intravenous techniques, including preprocedural infusion of paracetamol [32] or metamizole [33]. A reduction in the intraprocedural intravenous dose of OA administered in combination with GA has been proven to provide adequate analgesia postoperatively [28], with reduced incidences of PONV [23,26], despite potential side effects [3]. 

To the best of our knowledge, to date, no study has been performed to analyse the effect of either preventive regional or intravenous analgesia combined with GA and SPI-guided FNT administration on perioperative results in patients undergoing VRS. 

The aim of this study was to assess the effect of SPI-guided intraprocedural titration of the FNT dose on the cumulative dose of FNT, haemodynamic stability, and efficacy of postprocedural analgesia using the SPI and numeric pain rating scale (NRS) in patients undergoing VRS under GA alone or in combination with different techniques of preventive regional or intravenous analgesia.

## 2. Materials and Methods

Randomisation was performed by opening sealed envelopes in compliance with the Helsinki Declaration. Ethical approval for this study (KNW-1-183/N/9/K) was provided by the Ethical Committee of the Medical University of Silesia on 29th of September, 2015. The project was registered in the Clinical Trial Registry (SilesianMUKOAiIT2, NCT02973581). Study data can be accessed in the Department of Anaesthesiology and Intensive Therapy of the 5th Regional Hospital in Sosnowiec. The sample size was estimated at 200, given the total number of performed surgeries (average *n* = 500 per year and a half), a confidence level of 95%, and a margin of error of 5%.

Patients who were scheduled for elective primary VRS via the pars plana approach in the Department of Ophthalmology of the 5th Regional Hospital in Sosnowiec, Poland, and fulfilled the inclusion criteria were requested to participate in the study. We enrolled 200 patients with American Society of Anaesthesiologists (ASA) physical status class I–III after obtaining written informed consent.

Exclusion criteria were pregnancy, drug or alcohol abuse, a history of neurological disease or any neurosurgical operation that would impair entropy electroencephalography (EEG) monitoring, a history of pulmonary disease, anticipated difficult laryngeal mask airway (LMA) placement, acute or chronic pain, and cardiac arrhythmia on electrocardiography (ECG), which might impair SPI monitoring. 

Patients were randomly allocated into five groups: (1) Group GA, comprising patients who received general anaesthesia alone; (2) Group TA, comprising patients who received preventive topical analgesia by triple instillation of 2% proparacaine (Alcaine, propacaine hydrochloride ophthalmic solution, USP 0,5%, 15 mL, Sandoz a Novartis Company) 15 min before the induction of GA; (3) Group PBB, comprising patients who received PBB using a mixture of 3.5 mL each of 2% lignocaine (Lignocainum Hydrochloricum WZF 2% solution, 20 mg/mL, 2 mL, Polfa Warszawa S.A, Warsaw, Poland) and 0.5% bupivacaine (Bupivacainum Hydrochloricum WZF 0.5%, 5 mg/mL, 10 mL, Polfa Warszawa S.A, Warsaw, Poland) via Hamilton’s technique, 1 min before the induction of GA [34]; (4) Group M, comprising patients who received PA using a single dose of 1 g of metamizole (Pyralgin 0.5 g/mL, 5 mL solution; Polpharma SA, Starogard Gdański, Poland) in 100 mL of saline solution intravenously 30 min before arrival at the operating room; and (5) Group P, comprising patients who received PA using a single dose of 1 g of acetaminophen (Paracetamol Kabi 10 mg/mL, solution 100 mL; Fresenius Kabi, Warsaw, Poland) in 100 mL of saline solution intravenously 30 min before arrival at the operating room.

On the day of surgery, all patients were premedicated with 3.75–7.5 mg midazolam (Dormicum Midazolam 7.5 mg, Roche Polska Sp Z O. O., Warsaw, Poland) before the induction of anaesthesia according to body weight and age [35]. Before commencement of surgery, patients were preoxygenated for 5 min with 100% oxygen and intravenously infused with 10 mL/kg body weight of Ringer’s lactate solution (500 mL solution, Fresenius Kabi, Poland). Anaesthesia was induced intravenously with fentanyl at 1 µg/kg body weight (Fentanyl WZF, Fentanyl citrate, 50 microgram/mL, 2 mL solution, Polfa Warszawa S.A, Poland) and etomidate (Etomidate Lipuro, 2 mg/mL, 10 mL, Braun, Germany) at 0.2–0.3 mg/kg body weight intravenously. After loss of consciousness, rocuronium was administered at a standard intravenous dose of 0.6 mg/kg (Esmeron, rocuronium bromide, 10 mg/mL, 5 mL, Fresenius Kabi, Warsaw, Poland) for neuromuscular blockade, followed by placement of an LMA. The exhaled carbon dioxide concentration (EtCO2) level was maintained at 35–37 mmHg after LMA placement and before commencement of surgery; the sevoflurane concentration was maintained at a level of approximately 35–45 on state entropy. 

Throughout anaesthesia induction and surgery, standard monitoring was conducted with close attention paid to vital parameters, including the non-invasive arterial pressure (NIBP), HR, standard ECG lead II, pulse oximetry (SaO2), fraction of inspired oxygen in the gas mixture, fraction of inspired sevoflurane (FiAA), fraction of expired sevoflurane, EtCO2, and minimal alveolar concentration of sevoflurane. The depth of anaesthesia was monitored via entropy EEG (state and response). Intraoperative analgesia was guided by the SPI, and neuromuscular blockade was monitored (Carescape B650, GE, Helsinki, Finland). 

In Stage 1, on admission of patients to the operating theatre, we placed the sensor of the entropy EEG (state and response) on their forehead, a pulse oximeter (SPI) on their finger contralateral to venous access, an NIBP cuff on their right arm, and standard ECG leads on their back according to the manufacturer’s suggestions. Subsequently, the baseline values were recorded. 

PBBs were performed by the same ophthalmologist (MK-B) with over 6 years of experience with the procedure, including at least 400 PBBs a year. The sensory block was confirmed based on abolition of the corneal reflex. 

In Stage 2, SPI values were noted from 5 min after laryngeal mask placement to the beginning of sterilisation of the orbit to calculate the mean SPI value and allow calibration of the SPI sensor. 

In Stage 3, or the intraoperative stage, the SPI score was monitored online and recorded at 1-min intervals. When a ∆SPI value of >15 points was reached above the mean SPI value from Stage 2, a rescue dose of 1 µg/kg of FNT was administered intravenously every 5 min until the SPI value decreased to the mean SPI value recorded in Stage 2. The procedure time of VRS was taken as the duration from speculum insertion to removal.

We assumed that the initial dose of FNT of 1 µg/kg would produce sufficient analgesia for insertion of the speculum. In addition, Gruenewald et al. [13] proposed a ∆SPI value of >10 points or absolute SPI of >50 points as predictors of inadequate analgesia. In other studies, absolute ∆SPI of >50 points alone was an indication for rescue analgesia [3]. In our study, we used the protocol of a ∆SPI value of >15 points compared to the mean value recorded in Stage 2 lasting at least 1 min as an indication for rescue analgesia. We used this threshold to avoid possible hazardous overdosing of FNT resulting from potential miscalculations of the SPI value because of its variations. 

Vitrectomies were performed by the same ophthalmic surgeon (AL-B) with over 10 years of experience with VRS, including over 400 vitreoretinal procedures per year. Eye globe preparation included three or four 23-gauge ports. Initial core vitrectomy was followed by peripheral vitreous removal with scleral indentation. The removal of epiretinal membranes and/or internal limiting membrane peeling was a next surgical step. If needed, 4 sorts of tamponades were applied: temporary heavy perfluorocarbon liquids, air, SF6, or silicon oil. All retinal brakes or degenerations were treated with laser photocoagulation. The incidence of intraoperative Ocular-Cardiac Reflex (OCR) was recorded, which is typically identified by a rapid decrease in HR by 20% from the baseline during ocular manipulations. If OCR occurred, the surgeon was asked to stop surgical stimulation; intravenous atropine 0.5 mg (Atropinum Sulfuricum WZF 1 mg/mL, 1 mL solution, Polfa Warszawa S.A, Poland) was administered in cases of persisting bradycardia. If persisting hypotension occurred, a single dose of crystalloid was infused intravenously in a dose of 5 millilitres per kilogram of body weight, and a single dose of 5 milligrams of ephedrine (Ephedrinum hydrochloricum WZF 25 mg/mL, 1 mL solution, Polfa Warszawa S.A,, Warsaw, Poland) if crystalloid infusion failed to increase the mean arterial pressure (MAP) to >65 mmHg. 

In Stage 4, or the postoperative stage, patients were shifted to the recovery room, and patient monitoring was continued, with the SPI, HR, systolic arterial pressure (SAP), MAP, diastolic arterial pressure (DAP), and SaO2 measured by the anaesthesiology team, blinded to group allocation. Along with postoperative haemodynamic parameters, the presence of adverse effects, such as PONV and allergic reactions, was monitored for each patient. These observations were made at the time of pain assessment for the first 24 h postoperatively. In cases of PONV, 4 mg of ondansetron (Ondansetron Accord, Accord Healthcare Limited, Devon, UK) was administered intravenously. Optylite solution at 5 mL/kg was infused in cases of a MAP of <65 mmHg. Oxygen was administered at the rate of 3 L/min through a nasal cannula. Patients were asked to assess their perception of pain intensity on the numeric pain rating scale (NRS) ranging from 0 (no pain) to 10 (maximum pain) every 10 min. In the case of an NRS score of >3, non-steroid anti-inflammatory drugs at the standard dose were administered according to contemporary guidelines of acute pain treatment issued by the Polish Society of Anaesthesiologists [12]. The SPI was monitored online, and mean SPI values were recorded at 1-min intervals (trends in software provided by the manufacturer). The NRS and SPI values were recorded for severe (NRS 7–10), moderate (NRS 4–6), and mild pain (NRS 0–3) perception intervals. Each patient was observed and monitored in the recovery room for at least 30 min before being transferred to the Department of Ophthalmology when the patient’s Aldrette’s score was ten points. Monitoring and data recording were then ceased. 

Statistical analyses were performed using MS Excel and STATISTICA 12 (StatSoft, Poland). Data are expressed as the mean ± standard deviation or median (interquartile range). The Shapiro–Wilk test was used to evaluate the normality of distributions. The analysis included one-way analysis of variance for multiple groups (ANOVA) or the Kruskal–Wallis test by ranks. Additionally, post hoc tests were carried out to confirm differences between groups. For nominal data, we used percentages and tested for equality of proportions. Relationships between nominal variables were assessed using the χ^2^ test of independence. Statistical significance was set at a *p*-value of <0.05. 

## 3. Results

Of the 176 patients recruited in this study, the final analysis was performed on 175 patients, including 97 (55.4%) women and 78 (44.6%) men. With 40 (20%) patients in each group, patients were divided into five groups: GA, M, PBB, P, and T. One patient was disqualified from VRS because of sudden heart rhythm disturbances with hypotension. Finally, with 35 (20%) patients in each group, the patients’ nominal data were analysed in these five groups. The detailed characteristics of patients’ anthropometric and health data are shown in Table 1. There were no significant differences among groups in terms of age, height, or weight. Patients with weight in the norm were most often registered in the M and P groups. There were no significant differences in the percentages of patients with diabetes.

Only 3 out of 175 patients included in the final analysis declared acute postoperative pain perception, comprising 2 (5.7%) from the T group and 1 (2.9%) from the P group. Therefore, further analyses involved tolerable and intolerable pain perceptions. In the study groups, the numbers of patients with PIPP, requiring additional postoperative pain treatment, ranged from 5 (14.3%) in the PBB group, to 6 (17.1%) in the P and GA groups, to 8 (22.9%) in the M and T groups, comprising altogether 33 (18.9%) patients, disregarding group allocation. There were no statistically significant differences among groups in terms of pain intensity on the NRS scale, despite the differences in postoperative pain perceptions (Table 2).

Across all groups, the intraoperative requirement of rescue FNT was 129.9 ± 108.2 mcg, ranging from 95.1 ± 101.3 mg in the PBB group to 95.7 ± 81.7 mg in the P group, 144.3 ± 102 mg in the GA group, 148.6 ± 120 mg in the T group, and 165.7 ± 116 mg in the M group. Significantly higher FNT values were registered in patients from the M group, as compared to the PBB group (Table 3).

During VRS (Stage 3), significantly lower mean SAP, MAP, and DAP were observed in the PBB group compared to the other groups, while the maximum and minimum SAP, MAP, and DAP were also observed to be lower in patients in the PBB group. However, significantly higher mean SAP and MAP values, as well as their minimum and maximum values, were registered in the T group during PACU (Stage 4) (Table 4).

## 4. Discussion

Different techniques of RA are gaining increasing popularity for the performance of VRS. Excellent postoperative analgesia is usually guaranteed by PBB without sedation, which is dependent on both the anaesthetic technique and the local anaesthetic solutions used [36,37]. According to numerous studies in the literature, in select subgroups of patients, intraprocedural unacceptable pain perception negatively influences patient satisfaction with performance of the surgery [36,38].

As a result, to ensure adequate immobilisation on the operating table for the surgeon’s comfort, GA is induced in some elderly patients with expected inability to cooperate properly during VRS under RA or to consent to RA alone, or those who present with contraindications to RA [22]. Therefore, an additional 20% cost incurred with GA compared to RA with monitored anaesthesia care (MAC) must be taken into consideration [39]. The addition of different techniques of PA to GA aims to minimise the intraoperative OA requirement and to reduce the unwelcome incidence of PIPP. 

Although the addition of different techniques of PA to GA is reported to reduce the necessity of rescue OA, unfortunately, it hardly leads to the possibility of its complete elimination. Considering all of the above, the main aim of the current study was to assess the utility of SPI guidance for intravenous OA administration using FNT in cases of intraoperative afferent nociceptive stimulation due to an incomplete effect of different PA. 

Volatile anaesthetics, which are usually administered during GA, are reported to blunt the haemodynamic response to nociceptive stimulation [11]. During surgical intraprocedural manipulation with different intensity, according to the stage of VRS, patients receiving volatile anaesthetics are prone to experiencing afferent nociceptive stimulation that may not necessarily be reflected in fluctuations of haemodynamic parameters. The anaesthesiologist may falsely not administer rescue OA, being misled by the absence of an arterial blood pressure or HR increase; this may lead to PIPP due to central sensitisation. SPI values are reported to vary in response to the intensity of afferent nociceptive stimulation [16]; SPI monitoring yields a better measurement of the nociception–anti-nociception balance in comparison to the monitoring of fluctuations of haemodynamic parameters, including HR and arterial blood pressure, in the optimisation of intraoperative OA administration [15]. Derivation of the SPI value from finger plethysmography, which is further displayed on the screen, makes its use simple and intuitive, eliminating time-consuming preoperative preparations. SPI guidance during GA was reported to result in rational titration of rescue doses of OA and a reduction in the cumulative dose of OA administered during GA [19]. Fluctuations in the SPI value after a bolus administration of intravenous rescue FNT were shown to enable the monitoring of its intraoperative titration [18]. Therefore, SPI guidance of the titration of rescue OA helps in monitoring the effectiveness of rescue bolus doses of FNT. Based on these studies, we hypothesised that the use of SPI guidance for OA administration, in conjunction with different PA techniques, could possibly result in improvement of perioperative outcomes with lower incidences of haemodynamic instability and PIPP. 

Upton et al. [21] utilised anti-nociception index (ANI) guidance to administer FNT intraoperatively during GA with sevoflurane for lumbar discectomy and laminectomy. They observed that a more objective, ANI-guided intraoperative FNT administration process resulted in decreased perception of pain intensity in the immediate postoperative period when compared to the standard practice of FNT administration based on observation of haemodynamic fluctuations and the anaesthesiologist’s judgement. In contrast, Wennervirta et al. [17] showed that the addition of RA to GA was more efficient in providing perioperative analgesia when compared to SPI-guided OA titration in patients who underwent GA combined with the brachial plexus block (BPB). Different techniques of PA were utilised to ensure a smooth postoperative recovery. Analgesia initiated before a nociceptive afferent surgical stimulation is considered to be more effective than analgesia induced afterwards, as it suppresses the afferent nociceptive barrage perioperatively; this is the concept of preventive analgesia [40]. The action of local anaesthetics (LAs) results from a reversible block of sodium channels, which prevents the propagation of painful afferent nerve impulses from the cornea, conjunctiva, and sclera [40]. In contrast, metamizole and paracetamol are the most widely used non-opioid analgesics. They have both central (inhibition of cyclooxygenase type 3 (COX-3)) and peripheral mechanisms of action [41,42,43,44,45,46], and they have proven their analgesic efficacy in the treatment of postoperative pain, even in a single dose administered as pre-emptive analgesia [47,48,49,50,51,52,53,54]. 

Although the addition of PBB to GA was reported to diminish the requirement for intraoperative rescue OA, techniques of PA produce potential complications [53]. In the current study, no adverse events were observed; nevertheless, PBB has been reported to result in transient vision impairment, which may be an undesirable, distressing experience for patients postoperatively [54], and TA has been reported to cause local allergic reactions [53,54]. After PBB, systemic LA toxicity was reported to be likely to induce cardiac arrhythmias, increases in mean arterial blood pressure and HR [3], or a severe decrease in systolic blood pressure [55]. Perioperative haemodynamic fluctuations are a subsequent risk factor for the destabilisation of atherosclerotic plaques, which may result in life-threatening cardiac and cerebrovascular events [10]. Central retinal vein occlusion [56], brainstem anaesthesia [57], transient complete visual loss and partial third nerve palsy [58], pulmonary oedema [59], ocular explosion [60], and generalised tonic–clonic seizures [61] have been reported following PBB due to LA toxicity. In the current study, no adverse events associated with metamizole or paracetamol were observed; nevertheless, their use is not free from potential side effects [52]. Metamizole, despite being a controversial drug since it incidentally induces agranulocytosis [50,62,63,64,65,66], anaphylaxis [67], and Kounis syndrome (coincidental occurrences of allergic reaction and acute coronary syndrome secondary to vasospasm) [68], is widely used in developing countries due to its cost effectiveness [12]. VRS may cause PIPP [12]: 56% of patients were reported to complain of eye pain after VRS [69], while 48% of patients requested an analgesic within 5 h postoperatively, and 27% of patients required OA. GA with PBB using 0.75% ropivacaine with 75 IU of hyaluronidase in a volume of 5 mL was found to be superior in preventing PIPP when compared to a volume of 1 or 3 mL, and 60% of patients receiving 5 mL experienced no PIPP at 1 h after VRS [25]. Ghali et al. reported reduced PIPP in patients receiving PBB in combination with GA compared to GA alone in patients undergoing VRS, with scleral buckling also reported [23]. PIPP, defined as a score of >7 on the visual analogue scale (VAS), was reported by 7% of patients in the PBB group and 30% of patients in the GA group. Rescue doses of tramadol and total diclofenac consumption administered for moderate pain perception (VAS of >4) were also significantly higher in the GA group compared to the group in which GA was combined with PBB. 

PBB efficacy—expressed as adequate globe akinesia—of 20%–60% was observed, depending on the LA mixture used [36]; hence, on average, half of PBBs may theoretically provide insufficient analgesia. This is because estimation of the sensory block by abolition of the corneal reflex may not always imply the absence of sensory perception of surgical manipulations. 

In the current study, in the case of the employment of intravenous PA as an alternative to RA techniques, as compared to the control group, no statistically significant differences were found in the rates of PIPP, demand for rescue FNT administration, and clinically relevant haemodynamic stability between the *p* and M groups. The findings in our study are contrary to the findings of some other studies evaluating the efficacy of COX-3 inhibitors on the rates of PIPP in comparison with control groups. In patients undergoing panretinal photocoagulation, intravenous infusion of 1 g of metamizole 40 min before the induction of GA resulted in a statistically significant reduction in pain perception associated with the procedure in comparison to the placebo control group [70]. The analgesic potencies of intravenous PA using paracetamol and metamizole at a dose of 1 g proved their similar efficacies for postoperative analgesia after retinal surgery in comparison to the control group [32]. The influence of paracetamol administration for PA preoperatively or upon emergence from GA in patients undergoing PPV was observed to produce lower pain scores in both paracetamol groups compared to the control group at recovery [33]. However, an anaesthetic modality in which rescue OA was administered based on observance of the haemodynamic stability and the anaesthesiologists’ intuition was adopted, which could have markedly impaired the final rates of PIPP. 

Considering all the above, we hypothesised that supplementation of PBB or TA with intraoperative intravenous OA during GA under SPI guidance might be necessary and beneficial only in cases of a partly or completely failed block [36,38]. This might be a key factor in the utility of SPI guidance for supplemental FNT administration, creating a modern approach to multimodal intraoperative analgesia, where rescue boluses of FNT are added only in the case of insufficient PA expressed by an increase in SPI values at a certain stage of surgical manipulations or at certain stages of VRS, like laser treatment or trocars insertion. Similarly, the introduction of ultrasound-guided, perineural stimulation-directed (dual-guidance) interscalene PBB increased its efficacy from 41.46% with the perineural stimulation technique to 80.43% with the dual-guidance technique [24]. We hoped that, similar to dual-guidance BPB, ultrasound-guided PBB combined with GA in patients undergoing VRS could theoretically improve the efficacy of PBB through more precise needle placement and observation of LA deposition at the target destination in the future [71]. This might, in the end, reduce the necessity of intraoperative rescue OA administration using SPI guidance, as in the current study the demand for rescue FNT was the lowest in the PBB group, though without statistical significance. We also hoped to find intravenous PA similarly effective to PBB, as a number of patients have contraindications to the performance of PBB, like the necessity of pharmacological treatment using antiplatelet drugs; in these patients, SPI guidance for FNT administration will play only an additional role in achieving expected goals.

Overall, in our study, and to our great surprise, we did not observe marked improvements in perioperative outcomes in patients receiving either regional or intravenous PA in addition to GA with SPI-guided rescue FNT administration, compared to those receiving GA alone, although a statistically significant difference in the dose of intraoperative FNT administered between the PPB and M groups was noted. 

Out of the 175 patients in our study included in the final analysis, 30 (17.1%) complained of moderate pain, while 3 (1.7%) reported acute pain perception in the immediate postoperative period in the recovery room. Interestingly, the performance of PBB, TA, or intravenous infusion of M or *p* did not result in a significant decrease in PIPP expressed by NRS values; PIPP was reported by five (14.3%), eight (22.9%), six (17.1%), eight (22.9%), and six (17.1%) patients in the PBB, TA, GA, M, and P groups, respectively, resulting in the necessity of additional analgesia according to the contemporary guidelines and patients’ specific needs. With the use of SPI-guided FNT administration during GA, the afferent nociceptive stimulus was reflected in intraoperative ∆SPI values of >15 points compared to the baseline value. Inadequate PA detected by SPI monitoring resulted in more efficient suppression of central sensitisation. As a result, the use of preventive PA with intravenous or regional techniques did not influence the incidence of PIPP, showing a similar outcome in all groups; this is contrary to our recent study finding, where despite SPI guidance for FNT administration, infiltrative anaesthesia using a mixture of 0.2% ropivacaine with FNT significantly reduced PIPP and postoperative demand for morphine in patients subjected to lumbar discectomy under GA, as compared to infiltrative anaesthesia using a mixture of 0.2% bupivacaine with FNT and the placebo group [72].

A similar result was observed by Bayerl et al. [27] with preoperative RBB using bupivacaine 0.5% and mepivacaine 1% in combination with GA in patients undergoing VRS. They induced GA with FNT and propofol and maintained anaesthesia using propofol and remifentanil administered under observation for haemodynamic parameters and according to the anaesthesiologist’s judgement. No advantage of combining RBB with GA compared to GA alone with analgesia in the early postoperative period was observed in their study; however, in the GA group, COX inhibitors or non-steroidal anti-inflammatory drugs were infused before emergence, which may have influenced the incidence of PIPP. This resembled techniques of intravenous PA in our study. Our study findings were also in concordance with the observations made by Vaideanu D et al. [73], as PA analgesia using paracetamol in their study did not lead to a significant reduction in pain intensity associated with panretinal photocoagulation when compared to the control group. 

Haemodynamic instability during GA constitutes a serious risk factor for the development of cardiac and cerebrovascular events [10]. During VRS (Stage 3), significantly lower mean SAP, MAP, and DAP were observed in the PBB group compared to the other groups, while the maximum and minimum SAP, MAP, and DAP were also observed to be lower in patients in the PBB group. Although such differences seemed to bear little clinical significance (on average, not more than 10 mmHg), and no complications were observed in the case of PBB, as well as in the case of other PA modalities in the current study, PA combined with GA to achieve stable haemodynamics is no longer justified when an alternative of GA with SPI-guided FNT administration is available. Although the use of SPI-guided intraoperative analgesia with FNT in all groups resulted in similarly stable haemodynamics during VRS, as a tendency towards life-threatening hypotension was not observed, it is advisable to refrain from PA for the sake of the patients’ safety when SPI monitoring is available [74]. 

Several limitations to the current study must be taken into consideration. First and foremost, difficulty in the quantification of PIPP as a subjective phenomenon may have interfered in the final results, despite the number of patients allocated into final analysis [75]. Second, a control group without SPI guidance was purposely not designed, because numerous such studies have already been conducted, and the findings have been well-established. Third, anaesthetic modalities based on the use of GA alone without any supplemental medication to prevent PIPP may be questionable. Fourth, some reviewers in the British region prefer the anaesthetic technique of total intravenous anaesthesia using remifentanil and propofol (target-controlled infusion based on Schnider’s protocol), but numerous ASA physical status class III patients with coronary disease, overweight body mass index, obesity, or diabetes mellitus (see Table 1) were excluded because of the risks of intraoperative hypotension and bradycardia. Nevertheless, the use of sevoflurane seemed to be more beneficial for patients owing to sevoflurane-induced preconditioning [76] in addition to postoperative hyperalgesia resulting from intraoperative remifentanil in the control group. Both can raise ethical concerns, although studies using such a methodology have been published. The rate of PIPP after discharge from the recovery room to wards, because our study involved monitoring NRS and SPI values in Stage 4, was not analysed. Patient arousal, including changes in position and coughing, was reported to markedly interfere with SPI monitoring [77], making such a comparison difficult to interpret. We did not verify the akinetic effect of PBB after the induction of GA, as the only aim was to produce a sensory block. Finally, we decided to analyse the rates of incidences of the oculocardiac reflex, PONV, and risk factors of occurrence of adverse events like PPP and PONV in relation to the anthropometric data of patients in the studied groups as additional studies because of the manuscript approaching the word count limit.

## 5. Conclusions

In conclusion, despite the addition of PBB, TA, M, or P to GA, and a statistically significant reduction in the dose of intraoperative rescue FNT administered based on observance of fluctuations in SPI values over the course of GA, no benefit in perioperative outcomes was observed. Contrary to numerous studies on the efficacy of PA using either regional or intravenous techniques in patients undergoing VRS, in the current study, rational intraoperative SPI-guided rescue FNT administration under GA enabled the titration of optimal analgesia and haemodynamic stability. It is probable that the suppression of central sensitisation resulted in similarly low incidence rates of PIPP in all studied groups. Therefore, we suggest that the risks of rare potential perioperative complications of the administration of regional or intravenous analgesia outweigh its potential benefits when FNT is administered intraoperatively under SPI guidance, so it is advisable to invest in modern SPI monitoring once, rather than risk unnecessary complications in every single patient in everyday practice, with the accompanying legal consequences.

## Figures and Tables

**Table 1:** Anthropometric data of the patients in the studied groups.

Anthropometric Data		Total *n* = 175 (100%)	GA Group *n* = 35 (20%)	M Group *n* = 35 (20%)	P Group *n* = 35 (20%)	PBB Group *n* = 35 (20%)	T Group *n* = 35 (20%)	*p*-Value
**The results of the one-way analysis of variance (ANOVA)/the Kruskal–Wallis test by ranks**								
Age X ± S M (Rk)	[years]	64.5 ± 11.7 66 (13)	65.1 ± 10.8 67 (9)	61.9 ± 11.9 63 (14)	66.1 ± 9.9 67 (8)	66.8 ± 12.1 69 (13)	62.7 ± 13.3 65 (14)	*p* = 0.25 ^a^ NS
High X ± S M (Rk)	[cm]	165.8 ± 8.7 165 (12)	166.9 ± 8.6 168 (14)	168 ± 7.4 170 (14)	163.4 ± 8.7 160 (12)	165.9 ± 8.3 164 (12)	164.7 ± 10.3 164 (18)	*p* = 0.18 ^a^ NS
Weight X ± S M (Rk)	[kg]	77.6 ± 15.9 75.5 (17)	83.4 ± 19.8 82 (20)	74.7 ± 14.9 74 (19)	74.1 ± 13.3 74 (22)	78.8 ± 16 75 (11)	77.1 ± 13.7 80 (21)	*p* = 0.19 ^a^ NS
BMI X ± S M (Rk)	[kg/m^2^]	28.3 ± 5.4 27.5 (6.4)	29.9 ± 6.6 28.4 (5.3)	26.4 ± 4.6 25.3 (5.4)	27.9 ± 5.3 27.6 (7.7)	28.6 ± 5.1 27.1 (4.4)	28.5 ± 4.9 28.4 (7.3)	*p* = 0.05 ^a^
**The results of the χ^2^ test of independence**								
Gender *n* (%)	Female	97 (55.4)	18 (51.4)	15 (42.9)	24 (68.6)	21 (60)	19 (54.3)	*p* = 0.26 ^b^ NS
	Male	78 (44.6)	17 (48.6)	20 (57.1)	11 (31.4)	14 (40)	16 (45.7)	
Diabetes Mellitus	Insulin-dependent	53 (30.3)	11 (31.4)	6 (17.1)	12 (34.3)	12 (34.3)	12 (34.3)	*p* = 0.45 ^b^ NS
	Insulin-independent	45 (25.7)	10 (28.6)	3 (8.6)	12 (34.3)	9 (25.7)	11 (31.4)	*p* = 0.11 ^b^ NS
**The results of the multiple proportions test**								
BMI *n* (%)	Norm	50 (28.7)	5 (14.3)	15 (42.9)	14 (40)	7 (20.6)	9 (25.7)	*p* < 0.05 ^c^
	Overweight	72 (41.4)	18 (51.4)	13 (37.1)	9 (25.7)	19 (55.9)	13 (37.1)	*p* = 0.09 ^c^ NS
	Obesity	52 (29.9)	12 (34.3)	7 (20)	12 (34.3)	8 (23.5)	13 (37.1)	*p* = 0.41 ^c^ NS

Results are presented as means ± standard deviations and medians (interquartile ranges). ^a^ One-way analysis of variance (ANOVA)/the Kruskal–Wallis test by ranks. Nominal data are presented as numbers (percentages). ^b^ χ^2^ test of independence. ^c^ Multiple proportions test. BMI, body mass index. MAP—Mean Arterial Pressure; SAP—Systolic Arterial Pressure; DAP—Diastolic Arterial Pressure; PBB—preprocedural peribulbar block group; M group—metamizole group; P—Paracetamol group; X—mean; S—standard deviation; M—median; Rk—interquartile range; NS - statistically insignificant

**Table 2:** Rates of postoperative pain perception in patients regarding group allocation.

Scale	Total *n* = 175 (100%)	GA Group *n* = 35 (20%)	M Group *n* = 35 (20%)	P Group *n* = 35 (20%)	PBB Group *n* = 35 (20%)	T Group *n* = 35 (20%)	*p*-Value
NRS MAX X ± S M (Rk)	1.5 ± 2.1 0 (3)	1.5 ± 2 0 (3)	1.5 ± 2.2 0 (3)	1.6 ± 2.1 0 (3)	1.1 ± 1.9 0 (2)	1.8 ± 2.5 0 (3)	*p* = 0.84 ^a^ NS
Number of patients with postoperative acute pain perception—NRS > 6 *n* (%)	3 (1.7)	0 (0)	0 (0)	1 (2.9)	0 (0)	2 (5.7)	*p* = 0.25 ^b^ NS
Number of patients with postoperative moderate pain perception—NRS 4–6 *n* (%)	30 (17.1)	6 (17.1)	8 (22.9)	5 (14.3)	5 (14.3)	6 (17.1)	*p* = 0.88 ^b^ NS
Number of patients with postoperative mild pain perception—NRS ≤ 3 *n* (%)	140 (80)	29 (82.9)	27 (77.1)	28 (80)	30 (85.7)	26 (74.3)	*p* = 0.78 ^b^ NS
Number of patients with postoperative intolerable perception—NRS > 3 *n* (%)	33 (18.9)	6 (17.1)	8 (22.9)	6 (17.1)	5 (14.3)	8 (22.9)	*p* = 0.84 ^b^ NS
Number of patients unable to assess their postoperative pain perception *n* (%)	2	0	0	1	0	1	-

Results are presented as means ± standard deviations and medians (interquartile ranges). ^a^ One-way analysis of variance (ANOVA)/the Kruskal–Wallis test by ranks. Nominal data are presented as numbers (percentages). ^b^ Multiple proportions test. NRS, numeric pain rating scale; “-“—could not be statistically evaluated.

**Table 3:** Interoperative parameters.

Interoperative Parameters	Total *n* = 175 (100%)	GA Group *n* = 35 (20%)	M Group *n* = 35 (20%)	P Group *n* = 35 (20%)	PBB Group *n* = 35 (20%)	T Group *n* = 35 (20%)	*p*-Value ^a^
Time duration of VRS [min] X ± S M (Rk)	50.9 ± 18.9 47 (29)	47 ± 13.8 45 (22)	54.3 ± 20 57 (35)	48.2 ± 19.1 45 (30)	51.8 ± 23 42 (41)	53.1 ± 17.5 52 (25)	*p* = 0.47 NS
Interoperative requirement of rescue FNT [mcg] X ± S M (Rk)	129.9 ± 108.2 100 (150)	144.3 ± 102.7 150 (150)	165.7 ± 116.8 200 (200)	95.7 ± 81.7 100 (50)	95.1 ± 101.3 50 (150)	148.6 ± 120.3 150 (200)	*p* = 0.02 *p* < 0.05

Results are presented as means ± standard deviations and medians (interquartile ranges). ^a^ One-way analysis of variance (ANOVA)/the Kruskal–Wallis test by ranks. FNT, fentanyl; VRS, vitreoretinal surgery.

**Table 4:** Hemodynamic changes in patients with intraoperative pain perception during certain stages of anaesthesia.

Parameter	GA Group	M Group	P Group	PBB Group	T Group	*p*-Value ^a^
	*n* = 35 (20%)	*n* = 35 (20%)	*n* = 35 (20%)	*n* = 35 (20%)	*n* = 35 (20%)	
**Stage 1—ONSET**						
HR	73.6 ± 13.7	73.7 ± 12	73.3 ± 11.9	70.7 ± 12.1	69.4 ± 12.6	*p* = 0.38
(beats/min)	70 (20)	74 (19)	74 (17)	72 (19)	66 (17)	NS
SAP	152.7 ± 18	150.3 ± 17.8	150.1 ± 18.1	147.1 ± 24.3	158.8 ± 26.5	*p* = 0.48
(mmHg)	153 (25)	150 (30)	154 (22)	153 (35)	157 (35)	NS
MAP	110.7 ± 11.1	109.9 ± 12.3	108.2 ± 10.7	109.3 ± 12.8	113.4 ± 14.4	*p* = 0.47
(mmHg)	109 (17)	108 (21)	110 (13)	112 (24)	115 (26)	NS
DAP	79.5 ± 9	80.8 ± 10.4	76.6 ± 8.7	79.7 ± 9.1	81.1 ± 9.5	*p* = 0.28
(mmHg)	79 (12)	81 (15)	74 (15)	80 (10)	82.5 (16)	NS
SPI	53.1 ± 19.8	55.9 ± 18.8	54.3 ± 16.7	54.2 ± 20.1	54.6 ± 20.9	*p* = 0.9
	52 (29)	60 (29)	51 (19)	62 (34)	52.5 (29)	NS
**Stage 2—between LMA placement and start of VRS**						
mean HR	75.7 ± 14.5	68.2 ± 14.5	67.9 ± 10.4	69.5 ± 11.4	71.6 ± 14.5	*p* = 0.15
(beats/min)	76.5 (22)	71.8 (18.1)	66.8 (15.5)	67.5 (18.6)	73.4 (21.7)	NS
max HR	81.4 ± 15.3	74.7 ± 11.7	73.6 ± 12	75.3 ± 11.5	76.5 ± 13.7	*p* = 0.1
(beats/min)	82 (23)	76 (18)	73 (18)	74 (16)	79.5 (18)	NS
min HR	71.9 ± 13.3	67.3 ± 10.1	64.6 ± 9.9	64.8 ± 11.3	68 ± 14.4	*p* = 0.08
(beats/min)	73 (19)	69 (18)	64 (13)	64 (20)	67.5 (22)	NS
mean SAP	133.3 ± 26.3	124 ± 28.7	134.6 ± 24	121.3 ± 22.7	128.6 ± 23.2	*p* = 0.15
(mmHg)	136 (38.7)	129.5 (36.5)	134 (40)	118 (30)	125.2 (34.2)	NS
max SAP	145.3 ± 29.7	133.1 ± 30.4	142.2 ± 23.1	131.7 ± 21.2	140.8 ± 26.8	*p* = 0.24
(mmHg)	145 (42)	139 (39)	144 (43)	129 (22)	132 (44)	NS
min SAP	123.3 ± 28.5	115.8 ± 26.5	127.1 ± 26.9	112.7 ± 26.1	118.3 ± 23.3	*p* = 0.16
(mmHg)	116 (33)	116 (37)	133 (46)	105 (37)	118.5 (29)	NS
mean MAP	98.6 ± 17.3	95 ± 16.4	98.1 ± 15.6	90 ± 14.6	97 ± 17.3	*p* = 0.16
(mmHg)	97.5 (23.2)	99.7 (26)	100 (24)	87.5 (17.5)	93.8 (25.8)	NS
max MAP	106 ± 18.1	100.9 ± 17.8	103.1 ± 14.4	96.8 ± 13.5	103.9 ± 17.1	*p* = 0.17
(mmHg)	104.5 (21)	105 (22)	103 (21)	94 (16)	100.5 (25)	NS
min MAP	92.8 ± 21.9	89.6 ± 15.9	93.5 ± 17.9	83.9 ± 17.2	88.6 ± 15.8	*p* = 0.26
(mmHg)	90 (25)	90 (24)	92 (30)	82 (22)	86 (19)	NS
mean DAP	75 ± 13.1	72.8 ± 13.4	72.6 ± 11.2	68.4 ± 11.2	74.1 ± 10.3	*p* = 0.21
(mmHg)	73 (18.7)	74 (23.5)	72 (16.3)	68 (15.5)	74.7 (14.6)	NS
max DAP	79.6 ± 13.1	77.4 ± 14.5	76 ± 10.5	73.5 ± 10.5	81 ± 13.7	*p* = 0.11
(mmHg)	77 (19)	78 (23)	76 (15)	74 (17)	82 (16)	NS
min DAP	71.1 ± 14.6	69.4 ± 13.1	69.5 ± 12.9	64 ± 12.6	68.6 ± 10.8	*p* = 0.27
(mmHg)	69 (24)	67 (24)	67 (23)	63 (16)	68.5 (12)	NS
mean SE	45.1 ± 8.1	43.3 ± 8.1	42.7 ± 8.6	42.6 ± 8.7	46.8 ± 7.9	*p* = 0.17
	44.9 (13.5)	42.8 (14.3)	41.9 (13.2)	42 (13.5)	46.2 (12.7)	NS
max SE	50.2 ± 9.5	49.9 ± 8.1	48.4 ± 9.2	46.5 ± 9.8	52.6 ± 8.7	*p* = 0.28
	51 (16)	50 (12)	47 (15)	44 (16)	55 (12)	NS
min SE	41.1 ± 9.8	36.6 ± 7.6	35.5 ± 8.2	38.8 ± 8.6	40.5 ± 9.9	*p* < 0.05 GA vs. M * GA vs. P ** T vs. P *
	41 (14)	36 (11)	35 (14)	40 (10)	40.5 (15)	
mean SPI	34.6 ± 10.4	35.2 ± 13.8	39.4 ± 41.1	30 ± 7.1	30.8 ± 10.3	*p* = 0.05
	35.3 (14.8)	32.3 (18.8)	29 (16.7)	28.6 (9.1)	29 (14.5)	
max SPI	41.9 ± 12.3	43.3 ± 14.3	40 ± 11.8	37.4 ± 8.1	37.5 ± 11.5	*p* = 0.32
	41 (19)	41 (20)	36 (15)	36 (10)	37.5 (17)	NS
min SPI	28.3 ± 9.7	30.1 ± 13.5	27.2 ± 9.3	25.2 ± 7.6	26.1 ± 10.1	*p* = 0.54
	27 (12)	29 (14)	25 (10)	25 (12)	25 (12)	NS
**Stage 3—VRS**						
mean HR	68.6 ± 10.3	61.4 ± 7.1	59.5 ± 8.3	62.1 ± 9.1	64.6 ± 11.2	*p* < 0.01
(beats/min)	67.9 (17.2)	61 (11.2)	59.5 (11.5)	63 (13.9)	62 (16.1)	GA vs. P **
max HR	82.1 ± 13.7	73.5 ± 10.2	69.7 ± 10.7	74.7 ± 13.6	74.9 ± 13.8	*p* < 0.01
(beats/min)	81 (24)	72 (17)	69 (18)	74 (18)	72.5 (17)	GA vs. P **
min HR	61.2 ± 10.9	54.4 ± 7.5	53.4 ± 7.7	55.2 ± 8.6	57.2 ± 11.7	*p* < 0.05
(beats/min)	62 (14)	53 (12)	52 (8)	56 (13)	54.5 (15)	GA vs. P *
mean SAP	115.5 ± 22.7	107.5 ± 14	107.3 ± 23.3	105.9 ± 17.3	117.4 ± 16.5	*p* < 0.05
(mmHg)	108.1 (34.7)	108.4 (21.6)	104.2 (18.8)	100.7 (20.7)	117.4 (24.2)	PBB vs. T *
max SAP	149.3 ± 30.4	139.1 ± 30.1	137 ± 27.7	133.4 ± 27.8	144.6 ± 27.2	*p* = 0.13
(mmHg)	146 (48)	138 (37)	135 (37)	126 (39)	147.5 (32)	NS
min SAP	93.2 ± 18.5	86.1 ± 11.8	89.3 ± 15.9	87.5 ± 16.7	96.9 ± 14.2	*p* < 0.05 PBB vs. T * M vs. T *
(mmHg)	91 (29)	87 (18)	87 (21)	86 (17)	94 (20)	
mean MAP	87.5 ± 13.6	82.1 ± 10	81.8 ± 12.6	79.9 ± 11.6	88.4 ± 10.6	*p* < 0.01 GA vs. PBB ** GA vs. P * PBB vs. T **
(mmHg)	83.9 (23.3)	82.9 (15.8)	80.5 (14.7)	77.2 (11.9)	90 (15.1)	
max MAP	109.7 ± 19.5	104.2 ± 19.6	102.4 ± 18.8	99.6 ± 18.3	107 ± 16.2	*p* = 0.19
(mmHg)	108 (32)	102 (30)	99 (30)	94.3 (25)	108 (21)	NS
min MAP	71.1 ± 12.1	66.2 ± 9	67 ± 11.2	65.8 ± 11	73.7 ± 9.7	*p* < 0.01
(mmHg)	71 (19)	65 (11)	64 (16)	64 (15)	73 (12)	PBB vs. T **
mean DAP	66.1 ± 9.6	63.9 ± 8.5	62.1 ± 10.3	60.7 ± 8.8	68.1 ± 9.7	*p* < 0.01
(mmHg)	65.2 (14.5)	65.7 (9.5)	59 (11.2)	58.4 (12.6)	66.5 (10.7)	PBB vs. T **
max DAP	84.2 ± 14.9	81.9 ± 15.1	76.5 ± 14	75.4 ± 13.5	83.4 ± 12.1	*p* < 0.05
(mmHg)	79 (23)	83 (20)	74 (24)	75 (21)	83 (15)	A vs. PBB *
min DAP	54.7 ± 9.4	51.1 ± 8.6	50.7 ± 8	50 ± 7.9	57.1 ± 9	*p* < 0.01 PBB vs. T ** P vs. T *
(mmHg)	56 (13)	52 (13)	47 (12)	49 (14)	55.5 (13)	
mean SE	43.5 ± 5.7	41.1 ± 5	41.7 ± 7.5	45.6 ± 5.8	45.6 ± 5.2	*p* < 0.01 PBB vs. M * M vs. T *
	43.4 (8.2)	41.2 (6.8)	40.7 (10.8)	45.8 (7.4)	45.8 (7.6)	
max SE	55.2 ± 7.7	52.7 ± 7.1	55.4 ± 7.1	55.1 ± 5.9	54.9 ± 6.9	*p* = 0.47
	55.5 (12)	52 (13)	57 (9)	54 (9)	56 (11)	NS
min SE	34.9 ± 7.3	33.2 ± 6.6	32.1 ± 6.8	37.3 ± 7.3	37.8 ± 5.6	*p* < 0.001 PBB vs. P ** T vs. M * T vs. P **
	35 (9)	33 (9)	32 (8)	37 (9)	38.5 (6)	
mean SPI	33.9 ± 8.7	34.4 ± 10.9	34 ± 9.1	32.6 ± 7.1	33.5 ± 9.1	*p* = 0.99
	32.2 (10.3)	32.3 (12.8)	31.3 (9.8)	32.3 (8.7)	32.8 (13)	NS
max SPI	55.5 ± 11.9	57.2 ± 13.1	52.2 ± 11.2	53.7 ± 12.2	51.3 ± 13.7	*p* = 0.28
	53.5 (19)	56 (19)	55 (16)	53 (18)	50 (23)	NS
min SPI	22 ± 7.3	20.4 ± 7.7	22 ± 8	20.3 ± 6.7	20.9 ± 6.9	*p* = 0.74
	23 (11)	20 (9)	20 (9)	19 (9)	20 (10)	NS
**Stage 4—PACU**						
mean HR	74 ± 11.1	74 ± 12.4	68 ± 9.8	72.5 ± 11.4	70.4 ± 10.8	*p* = 0.13
(beats/min)	72.7 (16.1)	70.6 (13.5)	66 (13.9)	72 (12.9)	70.6 (17.8)	NS
max HR	80.3 ± 11.4	78.1 ± 13.5	72.2 ± 10.9	78.3 ± 11.3	77.1 ± 16.1	*p* = 0.08
(beats/min)	79 (15.5)	76 (17)	71 (16)	79 (10)	75 (19)	NS
min HR	69.5 ± 12.6	70.1 ± 11.6	64.8 ± 9	67.4 ± 11.4	66 ± 11.4	*p* = 0.37
(beats/min)	67 (19.5)	67 (14)	63 (13)	65 (16)	67 (17)	NS
mean SAP	152.4 ± 17.4	145.5 ± 14.6	146.4 ± 19	148.1 ± 18.2	158.8 ± 19.2	*p* < 0.05 M vs. T * P vs. T *
(mmHg)	152.4 (28.5)	146.8 (15.3)	143.2 (29)	146.3 (21.8)	156.7 (28.8)	
max SAP	164.2 ± 22.7	152.3 ± 16.4	153.1 ± 19.5	158.8 ± 21.7	167.2 ± 18.3	*p* < 0.01 M vs. T * P vs. T *
(mmHg)	163.5 (31)	154 (15)	154 (31)	152 (33)	166 (24)	
min SAP	145.7 ± 17.6	138.4 ± 13.3	140.7 ± 19.4	138.1 ± 18.3	152.8 ± 21	*p* < 0.05
(mmHg)	145.5 (27.5)	140 (24)	135 (33)	136 (15)	153.5 (31)	PBB vs. T *
mean MAP	107.7 ± 14.3	103.1 ± 13.9	101.7 ± 13.6	106 ± 10.1	113.7 ± 11.1	*p* < 0.01 M vs. T ** P vs. T **
(mmHg)	106.7 (14.6)	104.8 (15.3)	100.9 (18.3)	106 (12.7)	113.2 (13)	
max MAP	116.8 ± 14.7	110 ± 15	106.6 ± 13.5	114.1 ± 12.8	120.1 ± 10.8	*p* < 0.01 M vs. T * P vs. T ***
(mmHg)	114.5 (19)	109.5 (14)	106.5 (21)	113 (22)	119 (14.5)	
min MAP	103.7 ± 12.5	98.5 ± 14.4	98.8 ± 14.4	100.2 ± 13.8	108 ± 12.5	*p* = 0.05
(mmHg)	103 (18.5)	102 (18)	99 (16)	104 (20)	108.5 (17)	
mean DAP	77 ± 8.6	78.1 ± 10	75.6 ± 12.5	77.8 ± 8.3	81.8 ± 10.6	*p* = 0.11
(mmHg)	77.2 (11.3)	77.2 (12.3)	74.6 (15)	76.7 (11.7)	81.2 (15.7)	NS
max DAP	83.7 ± 9.8	83.1 ± 12.2	80.2 ± 12.7	84.3 ± 9.4	88.2 ± 14.3	*p* = 0.07
(mmHg)	83.5 (12.5)	81 (12)	77.5 (12)	83 (11)	89 (16.5)	NS
min DAP	72.9 ± 9.7	75.5 ± 10.6	71.4 ± 13.2	72.8 ± 9.4	77.8 ± 10.7	*p* = 0.06
(mmHg)	70.5 (13.5)	74 (14)	70 (13)	73 (10)	76.5 (16.5)	NS
mean SPI	51.3 ± 12.3	57.9 ± 17.6	53.6 ± 14.6	55.1 ± 13.5	54.1 ± 10.9	*p* = 0.4
	48.8 (12)	62 (32.8)	52.7 (21.5)	54.5 (21.9)	55.9 (15.6)	NS
max SPI	62.1 ± 12.1	66.2 ± 18	61 ± 14.8	63.9 ± 14.4	64 ± 10.6	*p* = 0.64
	59 (16)	70 (31)	58 (22)	62 (19)	63 (19.5)	NS
min SPI	41.6 ± 13.4	49.6 ± 16.3	46.6 ± 14.5	46.8 ± 13.5	44.8 ± 13	*p* = 0.2
	40 (14)	52 (30)	47 (22)	48 (21)	45 (16.5)	NS

Results are presented as means ± standard deviations and medians (interquartile ranges). ^a^ One-way analysis of variance (ANOVA)/the Kruskal–Wallis test by ranks. PBB—preprocedural peribulbar block group; M group—metamizole group; GA group—general anaesthesia group; T group—topical anaesthesia group; SPI—surgical pleth index; LMA—laryngeal mask airway; MAP—Mean Arterial Pressure; SAP—Systolic Arterial Pressure; DAP—Diastolic Arterial Pressure; HR—heartbeats; NS—non statistically significance; PACU—Post-Anaesthesia Care Unit; VRS—vitreoretinal surgery; “*”—statistically significance differences (*p* < 0.05); “**”—statistically significance differences (*p* < 0.01); “***”—statistically significance differences (*p* < 0.001).

## Data Availability

Study data can be accessed in the Department of Anaesthesiology and Intensive Therapy of the 5th Regional Hospital in Sosnowiec, Poland.
